# Bisphenol A Analogues Suppress Spheroid Attachment on Human Endometrial Epithelial Cells through Modulation of Steroid Hormone Receptors Signaling Pathway

**DOI:** 10.3390/cells10112882

**Published:** 2021-10-26

**Authors:** Hongjie Fan, Sudini R. Fernando, Luhan Jiang, Ziyi Wang, Suranga P. Kodithuwakku, Chris K. C. Wong, Ernest H. Y. Ng, William S. B. Yeung, Kai-Fai Lee

**Affiliations:** 1 Department of Obstetrics and Gynaecology, Li Ka Shing Faculty of Medicine, The University of Hong Kong, Hong Kong SAR, China; fan083@163.com (H.F.); sudini@uwu.ac.lk (S.R.F.); jessicaj@hku.hk (L.J.); wangziyi-cool11@163.com (Z.W.); suranga@hku.hk (S.P.K.); nghye@hku.hk (E.H.Y.N.); wsbyeung@hku.hk (W.S.B.Y.); 2 Department of Animal Science, Faculty of Agriculture, University of Peradeniya, Peradeniya 20400, Sri Lanka; 3 Department of Biology, Faculty of Science, Hong Kong Baptist University, Hong Kong SAR, China; ckcwong@hkbu.edu.hk; 4 Shen Zhen Key Laboratory of Fertility Regulation, The University of Hong Kong-Shenzhen Hospital, Haiyuan 1st Road, Futian District, Shenzhen 518053, China

**Keywords:** bisphenols, co-culture, spheroid attachment, steroid hormones, endometrium, microarray

## Abstract

Bisphenol A (BPA) is a well-known endocrine disruptor, widely used in various consumer products and ubiquitously found in air, water, food, dust, and sewage leachates. Recently, several countries have restricted the use of BPA and replaced them with bisphenol S (BPS) and bisphenol F (BPF), which have a similar chemical structure to BPA. Compared to BPA, both BPS and BPF have weaker estrogenic effects, but their effects on human reproductive function including endometrial receptivity and embryo implantation still remain largely unknown. We used an in vitro spheroid (blastocyst surrogate) co-culture assay to investigate the effects of BPA, BPS, and BPF on spheroid attachment on human endometrial epithelial cells, and further delineated their role on steroid hormone receptor expression. We also used transcriptomics to investigate the effects of BPA, BPS, and BPF on the transcriptome of human endometrial cells. We found that bisphenol treatment in human endometrial Ishikawa cells altered estrogen receptor alpha (ERα) signaling and upregulated progesterone receptors (PR). Bisphenols suppressed spheroid attachment onto Ishikawa cells, which was reversed by the downregulation of PR through PR siRNA. Overall, we found that bisphenol compounds can affect human endometrial epithelial cell receptivity through the modulation of steroid hormone receptor function leading to impaired embryo implantation.

## 1. Introduction

Bisphenol A (BPA), a well-known endocrine disruptor, is widely used in consumer products including plastics, paper bags, baby bottles, food cans, dental sealants, and thermal receipts [1]. Bisphenol A is ubiquitously found in air, dust, sewage leachates, and water. As a consequence, humans can be exposed to BPA through diet, inhalation of dust, and dermal contact [2]. As early as in the 1930s, BPA was found to have estrogenic effects on the female reproduction system [3]. It has been shown to bind to estrogen receptors and regulate gene expression [4]. In the past two decades, many studies have reported on the adverse effects of BPA, including reproduction, development, metabolic diseases, and the immune system in humans and laboratory animals [5,6,7]. As a result, several countries including Norway, Denmark, Germany, France, and the US have restricted the use of BPA in consumer products. They have been replaced with substitutes including bisphenol S (BPS) and bisphenol F (BPF), which have similar chemical structures to BPA.

Bisphenol A and bisphenol analogues, such as BPS and BPF, have been found in aquatic environments in several countries. Interestingly, levels of BPF were even higher than that of BPA in several Southeast Asian countries [8]. In seawater, BPF was found to be more biodegradable than BPA, but degradation of BPS was not observed under the same conditions [9]. Moreover, all three bisphenols have been detected in indoor air [10]. In foodstuffs, both BPA and BPF are found in beverages, dairy products, fish, meat products, fruit, and vegetables [11,12]. The amount of BPS in paper products and currency bills was found to be similar to BPA concentrations previously reported in thermal receipts [13,14]. In humans, urinary BPA was detected more frequently and at higher concentration than BPS and BPF [15,16].

Due to their structural similarity to BPA, recent studies have focused on the estrogenic effects of BPS and BPF [17,18]. It was reported that BPS and BPF can also interfere with the endocrine system in the humans. In vitro and in vivo studies showed both BPS and BPF had effects on hormone activation (estrogenic, anti-estrogenic, androgenic, and anti-androgenic) at a similar order of magnitude to that of BPA [17]. Interestingly, BPF was reported to bind estrogen receptors with similar potency to that of BPA, whereas BPS was less potent than BPA [18]. However, long-term low-dose BPS exposure in human osteosarcoma cells induced changes in the expression of more genes than BPA [19]. In a rat pituitary cell line, BPS disrupted estradiol-induced non-genomic signaling and altered cell proliferation, cell death, and prolactin release [20]. Maternal exposure to low-dose BPS impaired offspring development in zebrafish [21]. However, the effects of BPS and BPF on human reproductive health and embryo implantation are largely unknown, with only a few studies reporting that BPS and BPF increased uterine weight [22,23]. Besides estrogen receptors that are widely recognized as a receptor of BPA, another new receptor, GPR30, was recently identified [24]. The BPA analogues, BPS and BPF, have also been demonstrated to have estrogenic activities in regulating gene and protein expressions [25,26]. In this study, we used receptive endometrial Ishikawa cells to evaluate the effects of BPA, BPS, and BPF on endometrium receptivity, gene expressions, and embryo implantation using an in vitro co-culture assay.

## 2. Materials and Methods

### 2.1. Cell Culture

Endometrial adenocarcinoma Ishikawa cells (ECACC, 99040201) were maintained in Minimal Essential Medium (MEM, M0643, Sigma) supplemented with 10% fetal bovine serum (FBS, Invitrogen), L-glutamine, and penicillin/streptomycin at 37 °C in a humid atmosphere with 5% CO_2_. For the bisphenol studies, cells were cultured in Minimum Essential Medium without phenol red (M3024, Sigma), supplemented with 5% charcoal/dextran-stripped fetal bovine serum (csFBS, Gibco) before treatment. For spheroid generation, human choriocarcinoma Jeg-3 (ATCC, HTB-36) cells were maintained in DMEM/F12 (Sigma) with supplements.

### 2.2. Reagents

Bisphenol A (>99% purity, CAS 80-05-7), Bisphenol F (Bis(4-hydroxyphenyl) methane, 98% purity, CAS 620-92-8), and Bisphenol S (4,4′-Sulfonyldiphenol, 98% purity, CAS 80-09-1) were obtained from Sigma-Aldrich and were dissolved in DMSO. Methotrexate (MTX, Sigma) and 0.1% DMSO were used as controls in the experiments. Estrogen receptor antagonist (ICI 182,780), estrogen receptor α-specific antagonist (MPP dihydrochloride), estrogen receptor β-specific antagonist (PHTPP), and GPR30 antagonist (G15) were obtained from Tocris Bioscience. Lipofectamine 2000 (Invitrogen) was used for the cell transfection experiments. Luciferase expression was determined by Dual-Glo Luciferase Assay System kit (Promega). The MirVANA PARIS kit (Ambion) was used for RNA extraction.

### 2.3. Cell Proliferation Assay

Cells were seeded at 5 × 10^3^ cells per well in triplicate in 96-well plates in phenol red-free MEM with 5% csFBS. Cells were then exposed to different concentrations of BPA, BPF, and BPS for different time periods. DMSO at 0.1% and MTX at 5 μM were used as negative and positive controls, respectively. Total cell number was determined based on the cellular DNA content using CyQUANT Cell Proliferation Assay.

### 2.4. Cell Viability Assay

Ishikawa cells (3 × 10^5^) were seeded on a 6-well plate in MEM containing 10% FBS and supplements. The culture medium was changed to phenol red-free MEM without FBS to eliminate any potential estrogenic effects due to phenol red or hormones in FBS. After 24 h, cells were treated with 1–100 μM of BPA, BPF, or BPS for a further 24 h. Cells were trypsinized, stained with trypan blue, and counted using a hemocytometer.

### 2.5. Western Blotting

Total protein from BPA-, BPS-, and BPF-treated Ishikawa cells were extracted using mirVANA PARIS kit, and then separated by 8% SDS-PAGE and transferred to PVDF membrane. Western blot analysis was conducted with primary antibodies specific for ERα (HC-20, Santa Cruz), ERβ (EPR3778, Abcam), PR (M3569, DAKO), and GPR30 (ab39742, Abcam) from different sources, followed by anti-rabbit or anti-mouse secondary antibody conjugated with horseradish peroxidase (1:5000, GE Healthcare). After thorough washing, the membranes were visualized by enhanced chemiluminescence reagent (Santa Cruz). For protein normalization, membranes were stripped and reprobed with β-actin antibody (AC-15, Santa Cruz).

### 2.6. Transfection and Luciferase Assay

Ishikawa cells were seeded onto 12-well plates and cultured to 80–90% confluency before transfection. Cells were transfected with 1 μg luciferase reporter plasmid (3xERE-TATA-Luc) or control plasmid (pGL2-TATA-Luc) together with 0.1 μg internal control plasmid (pRL-TK) using lipofectamine 2000. Transfected cells were treated with different concentrations of bisphenols with or without steroid receptor antagonists. After 24 h, firefly and renilla luciferase activity were measured using Dual-Glo luciferase assay. The ratio of firefly to renilla luminescence in each well was calculated and compared with the empty vector controls.

### 2.7. RNA Extraction, RT-PCR, and Real-Time PCR

Ishikawa cells (3 × 10^5^) were seeded on 6-well plates in MEM with 10% FBS. Before treatment, the culture medium was changed to phenol red-free MEM without FBS for 24 h. Cells were treated with 10 μM BPA, BPS, or BPF with or without steroid hormone antagonists for 24 h. Total RNA was extracted and reverse transcribed using TaqMan reagents. The resulting cDNA was amplified using the TaqMan real-time PCR system, and the expression was normalized with the 18S internal control.

### 2.8. Microarrays

Total RNA was extracted as previously described [27], and RNA quality was analyzed by an Agilent 2100 Bioanalyzer (Agilent Technologies, Inc., Santa Clara, CA, USA). Microarray analysis was performed using a GeneChip Human Transcriptome Array 2.0 (Affymetrix) by The Centre for PanorOmic Sciences (CPOS) at Li Ka Shing Faculty of Medicine, University of Hong Kong. Per-chip normalization was performed using the robust multi-chip average (RMA) algorithm based on the expression values of all genes. The normalized expression values of all genes were then statistically analyzed by one-way ANOVA with *p*-value set at 0.01 or less. All differentially expressed genes (>2-fold and *p* < 0.01) were presented in a Venn diagram as upregulated and downregulated genes. Unsupervised clustering was employed to analyze differences in the gene expression profile between treatment groups based on the normalized Microarray data of all genes.

### 2.9. Spheroid-Endometrial Cells Attachment Assay

Human choriocarcinoma Jeg-3 cells were trypsinized, and 3 mL of the cell suspension at 1 × 10^5^ cell/mL was transferred to 6-well plates in 1% BSA containing DMEM/F12 medium, and then rotated at 88 rpm for 16–18 h to generate spheroids. Spheroids with size ranging from 60 to 200 μm were selected and transferred onto a confluent monolayer of endometrial Ishikawa cells in a 12-well plate under a light microscope. Before co-culture, Ishikawa cells were starved in phenol red-free MEM medium supplemented with 5% csFBS for 24 h, and then treated with bisphenols for another 24 h. Spheroids on endometrial Ishikawa cells were co-cultured for 1 h at 37 °C in a humidified atmosphere with 5% CO_2_. Undetached spheroids were removed by centrifugation at 140 rpm for 10 min. The number of attached spheroids was counted, and the attachment rate was expressed as the percentage of attached spheroids over the total number of spheroids transferred (% adhesion).

### 2.10. siRNA Transfection

Ishikawa cells were seeded on 12-well plate and treated with BPA (10 μM), BPS (100 μM), BPF (10 μM), and DES (10 nM) for 24 h. The treated cells were transfected with PGR siRNA (J-003433-05, -06, -07 and -08, Dharmacon) or non-target siRNA (D-001810-02-05) at a final concentration of 25 nmol/L with Lipofectamine 3000 (L3000001, Thermo Fisher, Waltham, MA, USA). Briefly, PGR siRNA and non-target siRNA were diluted in 50 μL Opti-MEM (Thermo Fisher, 31985-070), and 3 μL Lipofectamine 3000 was diluted in 50 μL Opti-MEM. The diluted siRNA was mixed with Lipofectamine 3000 solution for 20 min incubation in room temperature. The siRNA-Lipofectamine 3000 mixture was added to cultured cells for 6 h. Transfection medium was changed to normal culture medium for 48 h, and the cells were used for co-culture and Western blotting.

## 3. Results

### 3.1. Effect of Bisphenols, BPA, BPS, and BPF on Cell Proliferation and Cell Viability

**Figure 1:**
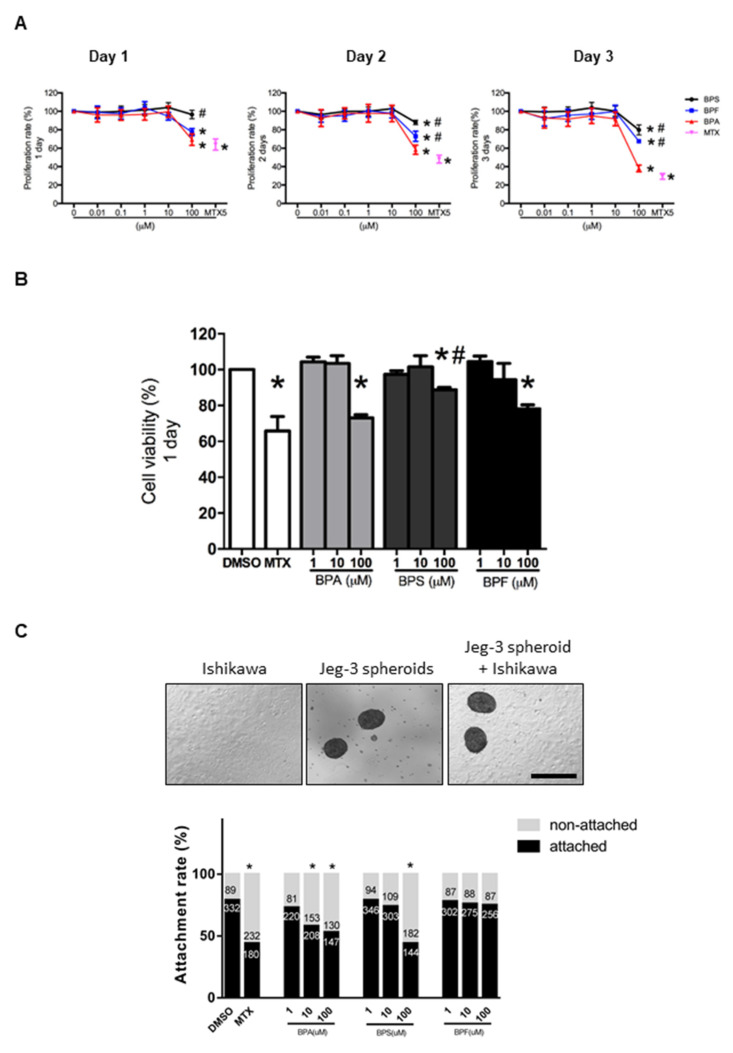
Effect of BPA, BPF, and BPS on Ishikawa cell proliferation, viability, and spheroid attachment. (**A**) The proliferation rate of Ishikawa cells treated with different concentrations of BPA, BPF, and BPS (0.01 to 100 μM) on days 1, 2, and 3 (*n* = 5). Methotrexate (MTX) at 5 μM was used as the control in the proliferation assay. (**B**) Cell viability of Ishikawa cells treated with different concentrations of BPA, BPF, and BPS (1 to 100 μM) on day 1 (*n* = 3). (**C**) Photomicrographs showing Jeg-3 spheroids, Ishikawa cells, and attached spheroids on Ishikawa cells. The attachment rate of Jeg-3 spheroids on Ishikawa cells treated with different concentrations of BPA, BPF, and BPS (1 to 100 μM) for 24 h prior. The number of attached spheroids over the total spheroids added is shown on the graph. * denotes *p* < 0.05 compared with DMSO control, and # denotes *p* < 0.05 compared with 100 μM BPA.

Human endometrial epithelial Ishikawa cells were exposed to 0.01–100 μM BPA, BPS, or BPF for 1 to 3 days. Cell proliferation was evaluated and compared with the control (0.1% DMSO). The high concentration (100 μM) BPA, BPS, and BPF, but not the lower concentrations, reduced cell proliferation from 1 to 3 days (Figure 1A). The reduction in proliferation was more prominent (*p* < 0.05) with 100 μM BPA on days 2 and 3 compared to BPF and BPS. Cell viability assessed by trypan blue staining showed 100 μM BPA, BPS, and BPF reduced the viability of Ishikawa cells after the 1 day treatment (Figure 1B). The decrease in cell viability was more prominent in BPA and BPF than in BPS. In all experiments, 5 μM MTX was used as the positive control, which significantly (*p* < 0.05) reduced proliferation and viability of Ishikawa cells.

### 3.2. Effect of BPA, BPS, and BPF on Spheroid Attachment Rate

We used Jeg-3 spheroids as an embryo surrogate in the in vitro model to study embryo-endometrium interactions. We showed that 10 and 100 μM BPA and 100 μM BPS significantly (*p* < 0.05) reduced spheroid attachment rate, whereas BPF had no suppressive effects on spheroid attachment even at 100 μM (Figure 1C). Again, 5 μM MTX was used as the positive control, which significantly (*p* < 0.05) suppressed spheroid attachment.

### 3.3. Effect of BPA, BPS, and BPF on the Expression of Bisphenol Receptors in Ishikawa Cells

**Figure 2:**
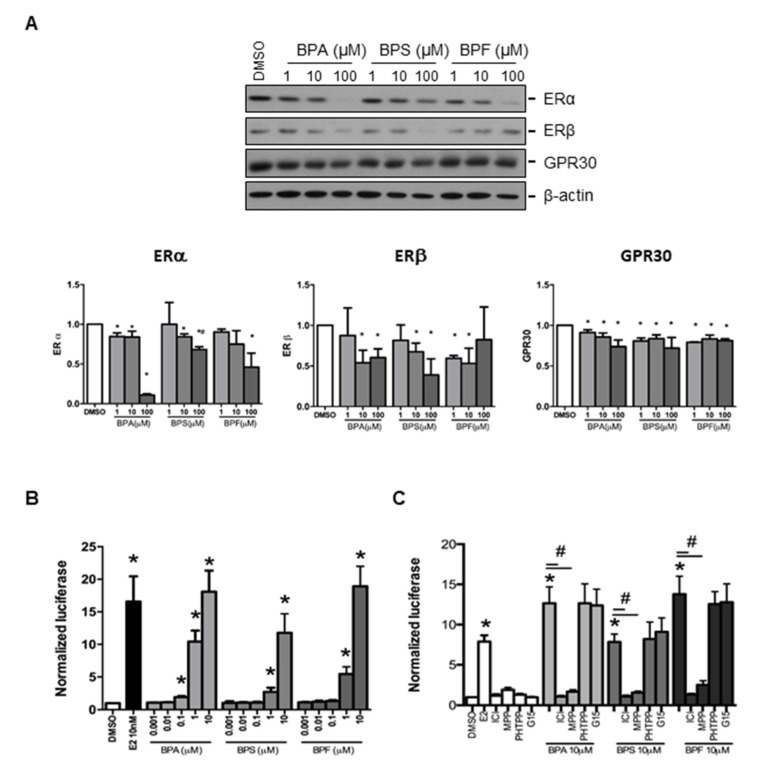
Effect of BPA, BPF, and BPS on the expression of ERα, ERβ, and GPR30 in Ishikawa cells. (**A**) Western blot analysis of the expression of ERα, ERβ, and GPR30 in Ishikawa cells treated with different concentrations of BPA, BPF, and BPS for 24 h (Top panel, *n* = 3). Semi-quantitative analysis of the Western blot images. (**B**) BPA, BPF, and BPS transactivated the 3xERE-TATA-Luc vector in transfected Ishikawa cells. Dual-Glo luciferase assay was used to detect the luciferase signal (*n* = 3). (**C**) Effect of ICI 182,780 (estrogen receptors antagonist), MPP dihydrochloride (estrogen receptor α-specific antagonist), PHTPP (estrogen receptor β-specific antagonist), or G15 (GPR30 antagonist) on the transactive effects of BPA, BPF, and BPS in transfected Ishikawa cells (*n* = 3). Estradiol at 10 nM was used as the positive control in all the transfection experiments, whereas DMSO was used as the solvent control. Signals were normalized to the Renilla signal in the co-transfected plasmid. Effect of ICI 182,780 (estrogen receptors antagonist) and G15 (GPR30 antagonist) on spheroid attachment on treated Ishikawa cells. DMSO and MTX (5 μM) were used as the negative and positive controls in the spheroid attachment assay. The number of attached spheroids over the total spheroids added is shown on the graph. * denotes *p* < 0.05 compared with DMSO and # denotes *p* < 0.05 compared with 10 μM bisphenol indicated in respective bars.

Estrogen receptors (ERα and ERβ) and GPR30 receptor were selected to study the effect of BPA, BPS, and BPF on regulating the expression of endometrial genes. We found that ERα, ERβ, and GPR30 proteins could be detected in Ishikawa cells by Western blotting (Figure 2A). Semi-quantitative analysis confirmed BPA, BPS, and BPF at 100 μM significantly downregulated ERα protein expression, with BPA showing the most significant decrease compared with BPF and BPS. Meanwhile, 10 and 100 μM BPA and BPS, and 1 and 10 μM BPF reduced ERβ expression. We also found that 1, 10, and 100 μM BPA, BPS, and BPF significantly (*p* < 0.05) reduced GPR30 protein expression (Figure 2A).

### 3.4. Effect of BPA, BPS, and BPF on the Regulation of Estrogen Responsive Element (ERE) Reporter Expression in Transfected Ishikawa Cells

We investigated the effects of BPA, BPS, and BPF on the classical (ERα and ERβ) and non-classical (GPR3) estrogen receptor signaling pathways. The 3xERE-TATA-Luc vector was first transfected into Ishikawa cells. We found 0.1 to 10 μM BPA and 1 to 10 μM BPF and BPS significantly (*p* < 0.05) activated luciferase expression (Figure 2B). Moreover, 1 and 10 μM BPA and BPF had higher transactivation activity on luciferase expression than BPS. The activation of ERE by 1 μM BPA was higher than that of 1 μM BPF. However, the transactivation activity of 10 nM estrogen was comparable to that of 10 μM BPA, BPS, and BPF, suggesting the estrogenic potency of BPA was 1000-fold less than estradiol. To further confirm the effects on estrogen receptor signaling pathway by bisphenols, estrogen receptor antagonist (ICI 182,780), estrogen receptor α-specific antagonist (MPP dihydrochloride), estrogen receptor β-specific antagonist (PHTPP), and GPR30 antagonist (G15) were used to study luciferase expression in transfected human endometrial Ishikawa cells. We found that ICI 182,780 and MPP, but not PHTPP and G15, antagonized the effects of BPA, BPS, and BPF on the estrogen receptor signaling pathway in transfected Ishikawa cells (Figure 2C).

### 3.5. Effect of BPA, BPS, and BPF on Progesterone Receptor Expression and Spheroid Attacment on Transfected Ishikawa Cells

**Figure 3:**
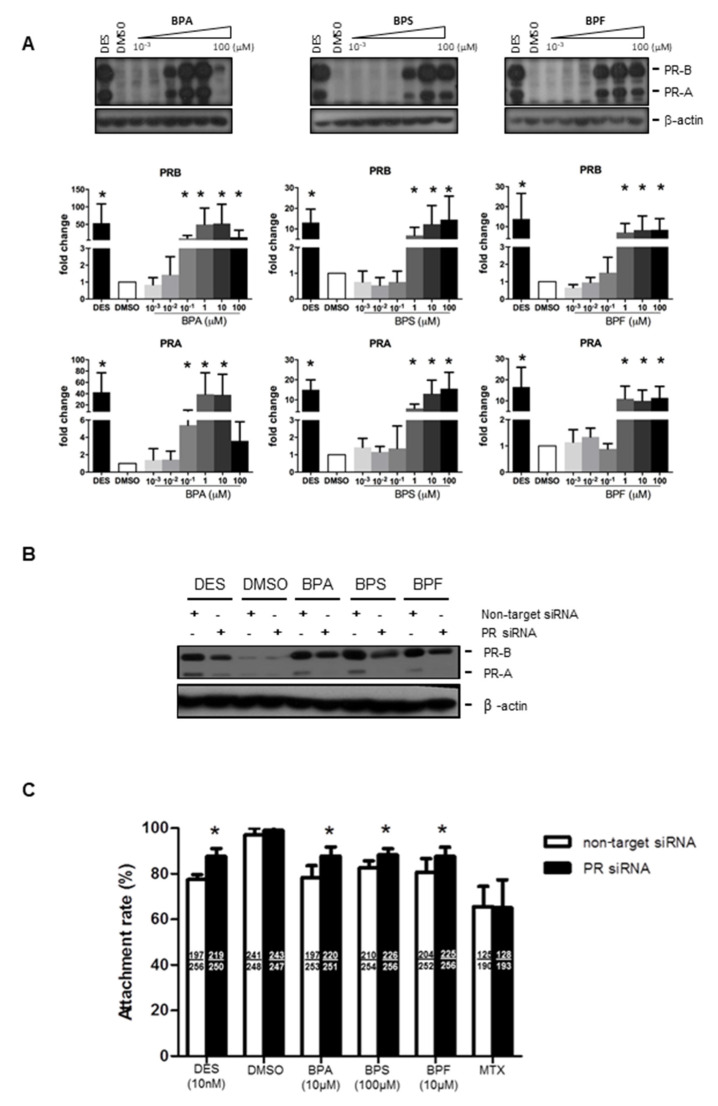
Effect of BPA, BPF, and BPS on the expression of PR-A and PR-B in Ishikawa cells and spheroid attachment. (**A**) BPA, BPF, and BPS (0.001 to 100 μM) treatment upregulated the expression of progesterone receptors PR-A and PR-B in Ishikawa cells. Western blot analysis demonstrated upregulation of PR-A and PR-B in treated Ishikawa cells (top panel). Semi-quantitative analysis demonstrated more than 10 to 50-fold increase in PR-A and PR-B expression in treated Ishikawa cells (*n* = 4). (**B**) PR siRNA downregulated PR-A and PR-B expression with BPA, BPF, and BPS treatment in Ishikawa cells. Western blot analysis demonstrated DES, BPA, BPS, and BPF, but not DMSO, upregulated PR-A and PR-B expression. PR siRNA suppressed the stimulation of PR expression by bisphenols in Ishikawa cells. (**C**) PR siRNA, but not non-target siRNA, reversed the suppressive effects on spheroid attachment by bisphenol-treated Ishikawa cells. DES was used as a control in the attachment assay. The number of attached spheroids over the total spheroids added is shown in each bar. * denotes *p* < 0.05 compared with non-target siRNA control.

We next examined if bisphenols also regulated progesterone receptor (PR) expression in Ishikawa cells. Progesterone receptor has two isoforms, PR-A and PR-B. We found that 0.1–100 μM BPA and 1–100 μM BPS and BPF upregulated PR-A and PR-B expressions in Ishikawa cells (Figure 3A). Similarly, the positive control diethylstilbestrol (DES, 10 nM), an estrogen agonist, was also able to upregulate PR expression in Ishikawa cells. The levels of PR upregulation were comparable between 10 nM DES and 1 μM BPA, BPS, and BPF, suggesting they have a strong potency (~100-fold) at higher concentrations (Figure 3A). We used PR siRNA to study the functional role of PR upregulation by bisphenol on spheroid attachment. Transfection of non-target siRNA did not change the expression of PR proteins. The strongly induced expressions of PR-A and PR-B by DES, BPA, BPS, and BPF were abolished by PR siRNA, but not by non-target siRNA, in the transfected Ishikawa cells (Figure 3B). No change in the house-keeping gene (β-actin) was found in all samples tested. Importantly, transfection of PR siRNA in Ishikawa cells reversed the suppressive effect on spheroid attachment by bisphenols and DES, but not MTX (Figure 3C).

### 3.6. Effect of BPA, BPS, and BPF on the Transcriptome of Treated Ishikawa Cells

**Figure 4:**
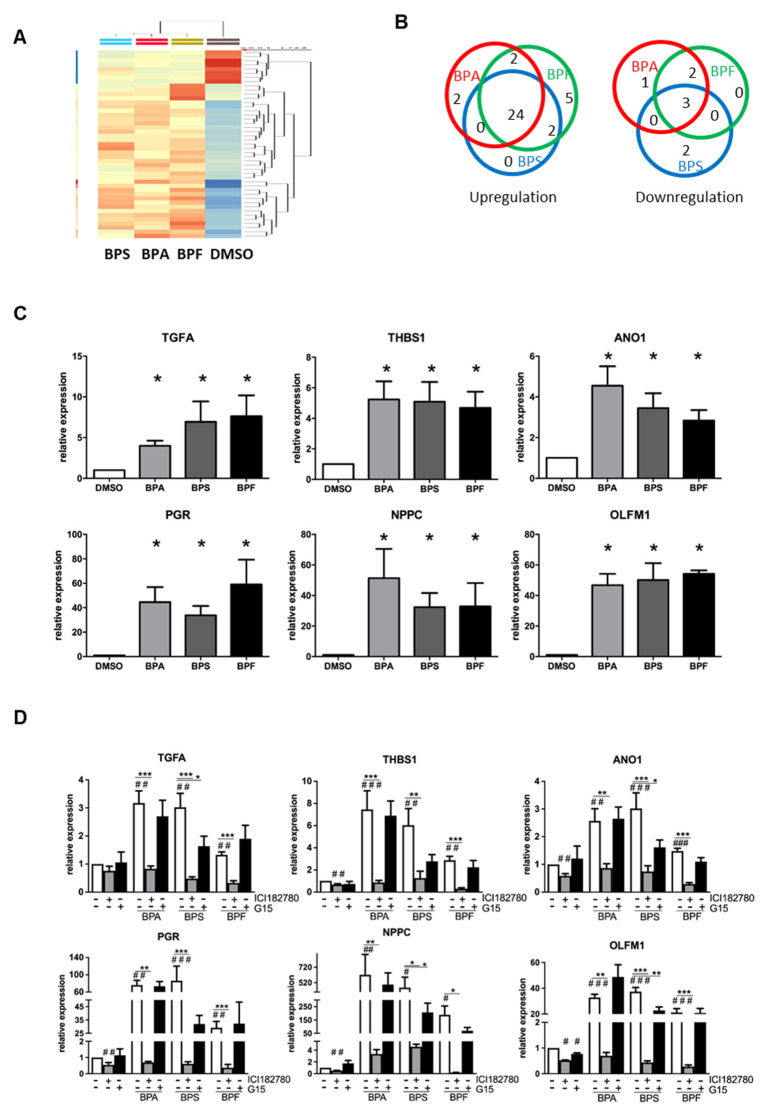
Microarray analysis of transcriptomic changes due to BPA, BPF, and BPS in Ishikawa cells. Ishikawa cells treated with 10 μM BPA, BPS, and BPF for 24 h were analyzed by GeneChip Human Transcriptome Array 2.0 (*n* = 3). (**A**) HeatMap clustering based on the similarity of regulated genes. (**B**) Venn diagram showing the number of differentially upregulated (left) and downregulated (right) genes in each treatment group compared with the DMSO control. Additionally, 24 out of 35 differentially upregulated genes and three out of eight differentially downregulated genes were found in all bisphenol treatments. (**C**) Six differentially expressed genes (TGFA, THBS1, ANO1, PGR, NPPC, and OLFM1) found in all bisphenol treatments were selected for qPCR validation (*n* = 7). * denotes significant differences from the DMSO control at *p* < 0.05. (**D**) Estrogen receptor antagonist (ICI 182,780 and G15) reversed the expressions of the six differentially upregulated genes induced by bisphenols. Ishikawa cells treated with 10 μM BPA, BPS, or BPF with or without 1 μM ER antagonists (ICI 182,780 and G15) for 24 h. qPCR analysis confirmed ICI 182,780 alone suppressed the expression of endogenous THBS1, ANO1, PGR, NPPC, and OLFM1 transcripts, and G15 also suppressed the expression of endogenous OLFM1 transcripts. ICI 182,780 suppressed induced TGFA, THBS1, ANO1, PGR, NPPC, and OLFM1 transcript expressions induced by 10 μM bisphenol. A significant suppression of OLFM1 expression was noted with G15 in BPS-treated Ishikawa cells. #, ## and ### denote significant differences from the DMSO control at *p* < 0.05, <0.01 and <0.005, respectively. *, ** and *** denote significant differences from the same bisphenol control at *p* < 0.05, <0.01 and <0.005, respectively.

To investigate if BPA, BPS, and BPF induce similar molecular changes in Ishikawa cells, we performed a transcriptomic analysis on bisphenol-treated Ishikawa cells. Total RNA of Ishikawa cells treated with 10 μM BPA, BPS, or BPF for 24 h were collected and analyzed by Microarray. Differentially expressed genes were selected based on statistical analyses (*p* < 0.05, one-way ANOVA) and on fold change (1.5-fold up-/downregulated) compared with the DMSO control. There was a total of 667 genes with *p* < 0.05, but only 43 genes had at least a 1.5-fold change in expression (Table 1). The clustering analysis in Figure 4A shows the high similarity gene expressions among BPA, BPS, and BPF treatment groups compared with the DMSO control. There were 35 upregulated and eight downregulated genes in the bisphenol treatments compared with the control (Figure 4B). The gene most upregulated by BPA, BPF, and BPS was progesterone receptor (PR or PGR), with 4.58-, 4.18-, and 3.74-fold increases, respectively. Of 35 upregulated genes, 24 were induced by all bisphenols, suggesting similar gene activation. Two genes (POLR3G and EPB41L2) were specifically upregulated (>1.5-fold) by BPA, and five genes (RNU7-24P, STC2, DNAJB9, and two novel transcripts) were specifically upregulated by BPF. Three genes (LGR5, GPR110 and SLC2A12) out of eight downregulated genes were suppressed by all bisphenols. Two genes (PIH1D2 and ERP27) were specifically downregulated by BPS, and two genes (MEOX1 and SOX4) were specifically suppressed by both BPA and BPF. The large overlap of genes regulated by all three bisphenols indicate that BPA, BPS, and BPF have similar effects on Ishikawa cells.

### 3.7. Gene Upregulation Is Mediated by ERs and Not GPR30

We validated the changes in the transcript levels by qPCR. We also further investigated the effect of ER antagonists (ICI 182,780 and G15) on bisphenol-regulated gene expression in Ishikawa cells. Based on the Microarray data (Table 1), six genes were selected, including the top three genes (PGR, THBS1, and NPPC) and three genes related to endometrial receptivity (ANO1, TGFA and OLFM1). Real-time PCR analysis confirmed the upregulation of these genes by BPA, BPS, and BPF in Ishikawa cells (Figure 4C). Interestingly, the fold changes were found to be higher than in the qPCR analysis. Putative estrogen responsive elements (ERE) were found in the upstream promoter regions of these genes. We found that 4, 1, 8, 1, 2, and 11 were putative ERE-sites at the promoter regions of the TGFA, THBS1, ANO1, PGR, NPPC, and OLFM1 genes, respectively, as determined by Dragon ERE Finder online (http://datam.i2r.a-star.edu.sg/ereV3/, 8 July 2016) (data not shown).

We further tested if the transcript expressions induced by bisphenols could be nullified by ER antagonists (estrogen receptor antagonist ICI 182,780 and GPR30 antagonist G15) in treated Ishikawa cell. We found the upregulation of six genes (TGFA, THBS1, ANO1, PGR, NPPC and OLFM1) by 10 μM BPA, BPS, and BPF was reversed by ICI 182,780 but not G15 (Figure 4D). However, G15 partially suppressed the upregulation of OLFM1 by BPS, which was still significantly higher than in the control group.

## 4. Discussion

In this study, we found low doses of BPA, BPS, and BPF did not affect cell viability or spheroid attachment on human endometrial epithelial cells. High doses of BPA, BPS, and BPF suppressed ERα, ERβ, and GPR30 expressions and induced PR expression in human endometrial epithelial cells. We found that BPA, BPS, and BPF acted through ERα to regulate the downstream signaling pathway. Treatment with PR siRNA nullified the suppressive effects of BPA, BPS, and BPF on spheroid attachment onto endometrial Ishikawa cells. Moreover, BPA, BPS, and BPF modulated similar subsets of genes in Ishikawa cells that control endometrial receptivity.

We first tested the effects of bisphenols on the viability of Ishikawa cells. High concentrations of bisphenol reduced the viability of Ishikawa cells, with BPA having the highest cytotoxicity among the three bisphenols tested. Bisphenols can act via the ER by binding to estrogen response element (ERE) to regulate gene expressions [28]. Studies showed that BPA can regulate the expression of some ERE responsive genes in vitro [29] through classical and non-classical estrogen signaling pathways in specific cell types [30]. Moreover, BPA mainly acts as an antagonist on estrogen receptors to exert its effect [31,32]. However, BPA can also disrupt the endocrine system through other hormone receptors, including thyroid hormone receptor [33,34] and androgen receptor [32,35].

The membrane estrogen receptor GPR30 was recently identified as a novel receptor for BPA [24,36]. It was shown that GPR30 can potentially be activated through non-classical estrogen pathways [24]. It contributes to estrogen physiology and pathophysiology in different contexts such as endometrium, pregnancy decidua, and implantation [37,38]. The estrogenic potencies of BPS and BPF have been compared with BPA in several cell lines, including MCF-7 cells and MELN cells [17]. Interestingly, the potencies of BPS and BPF on gene activation were found to be in the same order of magnitude as BPA.

Another method to investigate the estrogenic activity of bisphenols is to transfect ERE-TATA luciferase reporter plasmid into Ishikawa cells, which will be exposed to the bisphenols. Estrogen response element (ERE), a specific DNA sequence in the regulatory regions of some genes, is required for classical estrogen receptor binding and for regulating the expression of estrogen responsive genes [28]. In transfected Ishikawa cells, bisphenols activated estrogen responsive genes, as demonstrated by increased luciferase signals mediated by ERE binding, with BPA having higher potency than BPF and BPS. The gene activation by bisphenols in Ishikawa cells was mainly through ERα, because the increased luciferase signal was nullified only by ICI 182,780 (ERs antagonist) and MPP dihydrochloride (ERα-specific antagonist) but not PHTPP (ERβ-specific antagonist) or G15 (GPR30 antagonist). Similarly, BPA-induced gene activation in stromal cells of the uterus was inhibited by ICI 182,780 [39]. Induced cell proliferation by BPA in mouse Sertoli TM4 cells was shown to involve both GPR30 and ERα/β [40]. In human breast cancer cells, GPR30 was found to be necessary for BPA-induced activation of Erk1/2, cell proliferation and migration, and transcriptional regulation of genes (c-fos, EGR-1, and CTGF) independent of ERα/β-mediated signaling [36,41]. However, the effects and activity of BPA depend on the cell type and potential receptors [42]. As we showed, gene activation induced by bisphenols in Ishikawa cells involve ERE binding mediated through ERα but not ERβ or GPR30.

The progesterone receptor is a strong estrogen responsive gene, which has been found to be regulated by ERα [43]. Similar to our findings, other researchers showed that PR mRNA and protein expression was upregulated in BPA-treated Ishikawa cells [44]. In addition, BPA was reported to increase PR protein expression in the uterus of pregnant mice [45]. The progesterone-PR signaling pathway was found to be required and indispensable for the establishment and maintenance of pregnancy [46,47]. In mice, the expression of PR in luminal epithelial cells is increased from pregnancy days 2–4 but is extinguished on day 5 during the window of receptivity [48]. In humans, the downregulation of PR in endometrial epithelial cells was observed during pregnancy [49]. Therefore, BPA-induced expression of PR may disturb the normal dynamic expression of PR in the uterus, resulting in the aberrant activation of the PR signaling pathway, leading to compromised implantation and pregnancy.

We further investigated global transcriptomic changes in bisphenol-treated Ishikawa cells. The gene profiles of Ishikawa cells exposed to estrogen, DES, and BPA have been previously reported [50,51,52]. In this study, we investigated the transcriptomic changes due to BPA, BPF, and BPS in Ishikawa cell. Comparing a similar study by Naciff [52] and another study on Ishikawa cells exposed to BPA and DES [49], we identified the upregulation of TGFA, THBS1, PGR, and OLFM1 genes.

It was reported that OLFM1 (olfactomedin 1) is a negative factor for embryo implantation or pregnancy. A higher expression of OLFM1 was found in endometrial tissues from patients with unexplained recurrent spontaneous abortion [53]. An in vitro study found the spheroid attachment onto endometrial epithelial cells was suppressed by OLFM1 [54,55]. The expression of transforming growth factor alpha (TGFA) in human endometrial epithelial cells varies with menstrual cycle stage, with high expression levels together with high serum E2 levels in the late follicular and luteal stages [56]. However, the role of TGFA in pregnancy or embryo implantation is unknown. Thrombospondin 1 (THBS1) is an adhesive glycoprotein that mediates cell–cell and cell–matrix interactions and is an inhibitor of angiogenesis [57]. The expression of THBS1 is higher in the receptive phase than in the pre-receptive phase of human endometrial tissues. It was also shown to be highly expressed in receptive endometrial RL95-2 cells compared with non-receptive HEC1-A cells [58]. Moreover, the expression of THBS1 in Ishikawa cells was shown to be regulated by progesterone [59]. Decreased THBS1 expression in decidua macrophages was associated with unexplained recurrent spontaneous abortion [60]. Anoctamin 1 (ANO1) is calcium-activated chloride channel protein and was shown to be involved in myometrial contractility in human and murine myometrial tissue [61]. In mouse ovarian granulosa cells, estradiol production was enhanced by the inhibition or knockdown of ANO1 [62]. The role of ANO1 in endometrial function and pregnancy outcome still needs further investigation. Natriuretic peptide C (NPPC) is highly expressed in the uterus and placenta of mouse and human [63], and its receptor is also found in the uterus during pregnancy [64]. The expression of uterine NPPC was induced by estradiol in a mouse model [65]. An increase in myometrial NPPC expression was found to be associated with pregnancy complications (intrauterine growth retardation) [66], and an increased secreted NPPC level in the serum was found in women with complicated pregnancy [67]. However, the role of NPPC in endometrial receptivity and embryo implantation remains obscure.

Using different ER inhibitors (ICI 182,780 and G15), we found bisphenols acted through nuclear ER receptors, but not membrane ER receptors, to induce the expression of the six selected genes. This was confirmed by our luciferase reporter experiments, which showed that ICI 182,780 and MPP (ERα antagonist) nullified bisphenol-induced luciferase activity in the transfected Ishikawa cells. Similarly, bisphenol-induced luciferase activity has also been demonstrated in human breast cancer cells [68].

Several models have been established to study human embryo-endometrium interactions in vitro, including spheroids co-cultured with endometrial cell monolayer [69]. The receptivity of endometrial cells is critical for embryo implantation and pregnancy [70]. Previous studies identified genes that are changed in the human receptive endometrium [71]. With advances in single-cell sequencing techniques, the transcriptomic changes in the human endometrium at the single-cell level have now been reported [72].

In summary, we compared the effects of BPA, BPF, and BPS on endometrial Ishikawa cell toxicity, viability, spheroid attachment, and on the involvement of the ER signaling pathway. At high concentrations, BPA, BPS, and BPF downregulated ERα and stimulated PR expression. A similar finding was observed in human prostate cancer LNCaP cells, and the activation of EGFR/ERK/p53 signaling pathway was demonstrated after high doses of BPA exposure [73]. Knockdown of PR by siRNA reversed the suppressive effect of bisphenols on spheroid attachment, suggesting physiological doses of bisphenols may not affect human reproductive function in vivo. The transgenerational effects of bisphenols on human reproductive function warrant further investigation.

## Figures and Tables

**Table 1:** Genes regulated by 10 μM BPA, BPS and BPF in Ishikawa cell (fold change > 1.5, *p* < 0.05).

	*Transcripts Cluster ID*	*Gene Symbol*	*BPA*	*BPF*	*BPS*	*Gene Description*
***Upregulated in*** *BPA, BPF and BPF*	16743577	PGR	4.58	4.18	3.74	Progesterone receptor
*(24 genes)*	16799315	THBS1	3.43	3.71	3.84	Thrombospondin1
	16909551	NPPC	2.69	3.00	2.78	Natriuretic peptide C
	16660401	-	2.63	2.51	2.10	-
	16681827	DHRS3	2.45	2.81	2.87	Dehydrogenase/reductase (SDR family) member3
	16959386	SLCO2A1	2.34	2.11	1.81	Solute carrier organic anion transporter family, member2A1
	16728287	ANO1	2.33	2.39	2.69	Anoctamin1, calcium activated chloride channel
	16676983	G0S2	2.08	2.54	2.43	G0/G1 switch2
	16659940	-	2.05	1.86	2.03	-
	16898788	TGFA	2.04	2.18	2.21	Transforming growth factor, alpha
	16759324	MMP17	1.94	2.02	1.88	Matrix metallopeptidase17 (membrane-inserted)
	17118329	LOC100506966	1.92	2.15	1.88	-
	16851422	-	1.85	2.37	1.94	-
	16807268	-	1.85	2.08	1.84	-
	17048563	PEG10	1.85	1.78	1.65	Paternally expressed 10
	16692080	LOC100505987	1.81	1.69	1.71	-
	16691969	-	1.79	1.62	1.72	-
	17008092	PIM1	1.68	2.04	1.90	Pim-1oncogene
	16857389	NRTN	1.63	1.69	1.96	Neurturin
	17072669	MYC	1.63	1.73	1.61	v-myc myelocytomatosis viral oncogene homolog (avian)
	17072144	DEPTOR	1.62	1.72	1.82	DEP domain containing MTOR-interacting protein
	16829085	SLC7A5	1.61	1.60	1.61	Solute carrier family7 (amino acid transporter light chain, L system), member5
	16742244	GDPD5	1.54	1.64	1.83	Glycerol phosphodiester phosphodiesterase domain containing 5
	17091043	OLFM1	1.51	1.65	1.77	Olfactomedin1
*BPA and BPF*	16684192	SNORD99	1.51	1.66	1.39	Small nucleolar RNA, C/Dbox99
*(2 genes)*	16728329	-	1.54	1.63	1.45	-
*BPF and BPS*	16733038	-	1.39	1.64	1.55	
*(2 genes)*	16824901	SLC7A5P2	1.37	1.54	1.53	Solute carrier family7 (amino acid transporter light chain, L system), member 5 pseudogene 2
*BPA (2 genes)*	16987125	POLR3G	1.51	1.43	1.23	Polymerase (RNA) III (DNA directed) polypeptide G (32kD)
	17023551	EPB41L2	1.58	1.40	1.37	Erythrocyte membrane protein band 4.1-like2
*BPF (5 genes)*	16769861	-	1.16	1.63	1.15	-
	16825148	RNU7-24P	1.18	1.52	1.19	RNA, U7 small nuclear 24 pseudogene
	17002898	STC2	1.44	1.51	1.35	Stanniocalcin2
	17050328	DNAJB9	1.08	1.53	1.09	DnaJ (Hsp40) homolog, subfamily B, member 9
	17060057	-	1.27	1.61	1.14	-
***Downregulated in*** *BPA, BPF and BPF*	16754134	LGR5	−1.58	−1.64	−1.67	Leucine-rich repeat containing G protein-coupled receptor 5
*(3 genes)*	17019778	GPR110	−1.58	−1.73	−1.77	G protein-coupled receptor 110
	17023799	SLC2A12	−1.70	−1.64	−1.61	Solute carrier family 2 (facilitated glucose transporter), member 12
*BPA and BPF*	16845427	MEOX1	−1.55	−1.53	−1.29	Mesenchyme homeobox1
*(2 genes)*	17005276	SOX4	−1.61	−1.59	−1.42	SRY (sex determining region Y)-box4
*BPA (1 gene)*	17005197	RNF144B	−1.57	−1.44	−1.47	Ring finger protein 144B
*BPS (2 genes)*	16744393	PIH1D2	−1.44	−1.35	−1.56	PIH1 domain containing 2
	16761830	ERP27	−1.29	−1.44	−1.52	Endoplasmic reticulum protein 27

## Data Availability

All the data are available with the corresponding author and can be accessed with a valid reason.
